# Structural, Magnetic, and Magneto-Optical Properties of Thin Films of BaM Hexaferrite Grown by Laser Molecular Beam Epitaxy

**DOI:** 10.3390/ma16124417

**Published:** 2023-06-15

**Authors:** Boris Krichevtsov, Alexander Korovin, Sergey Suturin, Aleksandr A. Levin, Ivan Lobov, Andrey Telegin, Andrey Badalyan, Vladimir Sakharov, Igor Serenkov, Maxim Dorogov, Nikolai Sokolov

**Affiliations:** 1 Ioffe Institute, Politechnicheskaya 26, 194021 St. Petersburg, Russia; boris@mail.ioffe.ru (B.K.); amkorovin@mail.ioffe.ru (A.K.); suturin@mail.ioffe.ru (S.S.); andrey.badalyan@mail.ioffe.ru (A.B.); vlad.sakh@rambler.ru (V.S.); r.ilin@mail.ioffe.ru (I.S.); nsokolov@fl.ioffe.ru (N.S.); 2 M.N. Mikheev Institute of Metal Physics, 18 S. Kovalevskaya Str., 620108 Ekaterinburg, Russia; i_lobov@imp.uran.ru (I.L.); telegin@imp.uran.ru (A.T.); 3 Institute of Advanced Data Transfer Systems, ITMO University, Kronverksky Pr. 49, 197101 St. Petersburg, Russia; mvdorogov@itmo.ru

**Keywords:** hexaferrite, molecular beam epitaxy, crystal structure, hysteresis loops, XRD, magneto-optical Kerr effect, VSM, FMR

## Abstract

Thin films of BaM hexaferrite (BaFe_12_O_19_) were grown on α-Al_2_O_3_(0001) substrates by laser molecular beam epitaxy. Structural, magnetic, and magneto-optical properties were studied using medium-energy ion scattering, energy dispersive X-ray spectroscopy, atomic force microscopy, X-ray diffraction, magneto-optical spectroscopy, and magnetometric techniques, and the dynamics of magnetization by ferromagnetic resonance method. It was shown that even a short time annealing drastically changes the structural and magnetic properties of films. Only annealed films demonstrate magnetic hysteresis loops in PMOKE and VSM experiments. The shape of hysteresis loops depends on thickness of films showing practically rectangular loops and high value of remnant magnetization (*M*_r_/*M*_s_~99%) for thin films (50 nm) and much broader and sloped loops in thick (350–500 nm) films. The magnitude of magnetization 4π*M*_s_ ≈ 4.3 kG in thin films corresponds to that in bulk BaM hexaferrite. Photon energy and sign of bands in magneto-optical spectra of thin films correspond to ones observed earlier in bulk samples and films of BaM hexaferrite. FMR spectra of 50 nm films at 50 GHz consist of a number of narrow lines. The width of main line ΔH~20 Oe is lower than has been reported up to now.

## 1. Introduction

One of the fundamental limitations hindering the increase in the speed and volume of information transmission and processing is the release of Joule heat during the transport of charge carriers. A possible way to solve this problem is by the utilization of magnonic devices based on the use of spin waves packets propagating in magnetic nanoheterostructures [1,2,3]. In this regard, the problem of creating thin film materials in which it is possible to excite, control, and record weakly damping spin waves arises. For these purposes, intensive studies of nanostructures based on garnet ferrites [4,5,6,7,8,9], spinel ferrites [10,11,12,13,14], and orthoferrites [15,16,17] were carried out.

Desirable parameters for the practical application of magnonic structures are out-of-plane orientation of the magnetization without an external magnetic field, a small value of the ferromagnetic resonance (FMR) line width, and a high value of the magnetic moment, which means a large value of the film thickness [18]. As previous studies show, it is difficult to fulfill all these conditions. Thin films of ferrite garnets, in particular, yttrium iron garnet, which have record-breaking minimum FMR line widths, are characterized by low values of the magnetic anisotropy field and demonstrate a planar orientation of the magnetization in the absence of a magnetic field [5,6,7,8]. Ferrimagnetic spinels, in particular Ni ferrite (NFO), have higher values of magnetization and magnetic anisotropy field but also exhibit in-plane orientation of magnetization and higher values of FMR line widths [10,11,12].

Thin films of ferrimagnetic hexaferrites and, in particular, Ba-hexaferrite of M-type (BaM, BaFe_12_O_19_), are currently attracting much attention due to the unique magnetic parameters of this material. A review of the synthesis, properties, and applications of hexaferrites can be found in ref. [19]. Compared with the above-mentioned magnetic garnets, spinels and orthoferrites, hexaferrites have a number of advantages. The magnetization of BaM hexaferrite at room temperature (RT) 4π*M*_s_~4 kG is higher than in yttrium iron garnet (YIG) 4π*M*_s_~1.7 kG and nickel ferrite 4π*M*_s_~3.3 kG. The uniaxial magnetic anisotropy field *H*_a_ in BaM hexsaferrite is *H*_a_ ≅ 1.75 T, that is two orders of magnitude higher than in YIG and one order higher than that of NFO. Due to the high value of *H*_a_, devices based on hexaferrites can operate at frequencies up to *f* = 60 GHz. Moreover, textured polycrystalline hexaferrites can be created with a significant remnant magnetization, which in some cases makes it possible to avoid the use of external magnets.

For these reasons, a number of works have recently been carried out on the fabrication of thin films of BaM hexaferrite by various methods and the study of their magnetic properties. The results of these works can be found in review articles [18,19,20]. Thin films of hexaferrites were fabricated by pulsed laser deposition (PLD) [21,22,23,24,25], liquid phase epitaxy (LPE) [26,27,28], direct current magnetron sputtering [29], screen printing (SP) [30], and metallo-organic decomposition (MO) [31] methods on different substrates (sapphire, Al_2_O_3_, MgO, GdGa-garnet (GGG), 6H–SiC). Comparison of films prepared by different methods, presented in [18], shows that the films of high crystal quality, prepared by PLD or LPE, show an out-of-plane orientation of *c*-axis and small values of FMR lines widths (~30–60 Oe) but also small values of remanence *M*_r_ for out-of-plane hysteresis loops. For this reason, for the application of these films in microwave devices an external magnetic field is needed. Contrary to that, the films of lower crystal quality, fabricated by SP [30], have high hysteresis loop squareness (*M*_r_/*M*_s_~0.9), showing realization of “self-bias” effect, but large values of FMR line width. Thus, obtaining hexaferrite films with high crystal quality and high self-bias values is very desirable. Note that epitaxial films of BaM hexsaferrite with in-plane orientation of easy axis and “self bias” effect were synthesized by direct current magnetron sputtering on α-plane (11–20) of single-crystal sapphire substrates [29].

Intensive studies of films and nanostructures based on hexaferrites continue to the present. Recent papers on this topic, in particular, present the results of studying strontium hexaferrite (SrFe_12_O_19_) films obtained by pulsed laser deposition [32], the effect of La substitution on the structural and chemical properties of barium hexaferrite [33], the magnetic properties of nanostructured BaFe_12_O_19_ synthesized by sol–gel spontaneous combustion [34], mechanical regulation magnetic properties of uniaxial anisotropic thin films of hexaferrite [35] etc. The main objectives of presented article were fabricating epitaxial thin (with thickness *h* = 50–500 nm) films of hexaferrite BaM (BaFe_12_O_19_) grown by laser molecular beam epitaxy on α-Al_2_O_3_(0001) substrates, studying the chemical composition, surface morphology, crystal structure, static and dynamic magnetic properties, and magneto-optical spectroscopy. Much attention is paid to the comparison of composition, structural, and magnetic properties of as-grown and annealed structures and dependence of magnetic properties on film thickness.

## 2. Materials and Methods

Thin films (thickness *h*~50–500 nm) were grown using the laser molecular beam epitaxy (LMBE) method on α-Al_2_O_3_ (0001) substrates with an installation produced by Surface GmbH (Hückelhoven, Germany). The films were grown in an oxygen atmosphere at a pressure of *p* = 0.04–0.06 mBar at growth temperatures of *T*_gr_ = 750 °C–1000 °C. The flow of matter onto the substrate was created by ablation of a stoichiometric BaFe_12_O_19_ (BaM) target by a KrF excimer laser with a wavelength of 248 nm. The target was fabricated according to the standard technology for the manufacture of ferrites from a charge containing oxides BaCO_3_ and Fe_2_O_3_. To determine the best growth parameters, more than 25 structures were grown in various modes, i.e., at various growth temperatures, oxygen pressures, pulse repetition rates, and various annealing modes. For our study, we used both the as-grown structures and structures after annealing procedure. For post-growth annealing, the samples after preparation were removed from the growth chamber and annealed up to 4 h (240 min) in air at the temperature of 1000 °C. Table 1 shows growth and annealing parameters of the samples (single-layer structures) presented in this paper.

The chemical composition of the BaM films was studied using energy dispersive X-ray spectroscopy (EDX) (Ultim MAX 100 with AZtec software, Oxford Instruments,Oxford, UK) on a TESCAN scanning electron microscope (SEM) (TESCAN ORSAY HOLDING, a.s.; Brno-Kohoutovice, Czech Republic) with a spectrum analyzer attachment (Oxford Instruments plc, Abingdon, Oxford, UK). The analysis was carried out at the 20 keV energy range and 800,000 count limit. The measurements were carried out on annealed and unannealed samples of 50 nm thick. On each sample, the EDX spectra were measured at four points.

The chemical composition of the films was also studied by medium-energy ion scattering (MEIS) method, which makes it possible to study nanometer-thick films of various compositions and, in particular, to determine the film thickness and its inhomogeneity, elemental composition, and its depth distribution in a non-destructive way. The method includes recording the energy spectra of backscattered ions (BSI) and determining the parameters of the film based on a comparison of the measured spectra with the calculated or standard ones obtained in the study of films of a known composition. The films were probed with H^+^ and He^+^ ions with an energy of 227 keV. BSI spectra were recorded by electrostatic analyzer, providing the depth resolution in the near-surface region of 0.5–1 nm.

To characterize the surface morphology and crystal quality of grown films, the Al_2_O_3_(0001) substrates and films grown on them were monitored using an NT-MDT atomic force microscope (NT-MDT LLC, Zelenograd, Russia) in the semicontact mode at RT. Atomic force microscopy (AFM) measurements were carried out both for the as-grown and annealed single-layer structures, as well as for films obtained under various growth conditions.

The growth of a hexaferrite layer on the substrate surface was controlled by reflection high-energy electron diffraction (RHEED). The crystal structure of the grown layers was studied in situ using three-dimensional (3D) mapping of the diffraction intensity distribution. To do this, a series of RHEED patterns were taken during rotation of the sample about the normal to the surface. As a result of *φ* scanning with a step of 0.5° in *φ*, a series of 900 images was measured, from which a 3D map of the reciprocal space was obtained using the software. This made it possible to obtain a 3D distribution of the diffraction intensity in the reciprocal lattice and the projection of the intensity distribution onto the chosen reciprocal lattice plane. To analyze the RHEED patterns, the method of 3D mapping of diffraction patterns was applied.

X-ray diffraction (XRD) measurements were carried out using desktop powder X-ray diffractometer D2 Phaser (Bruker AXS, Karlsruhe, Germany) constructed in vertical Bragg–Brentano (reflection) *θ*-*θ* geometry and supplied with linear semiconductor position-sensitive detector LYNXEYE and Cu-*K*_α_ radiation (wavelength *λ* = 1.5418 Å) of X-ray tube with copper anode filtered with Ni-foil filter. For the XRD measurements, the film samples were placed at low-background single-crystal Si(119) sample holder. To reduce the influence of effects of preferential orientation, during the measurements, the samples were rotated around the axis coinciding with the axis of the goniometer of the diffractometer. Corrections for zero shift Δ2*θ*_zero_ and displacement Δ2*θ*_displ_ to obtain corrected Bragg angle values 2*θ*_B_ [36] were determined based on additional XRD measurements of samples immersed in NaCl powder calibrated using XRD standard powder Si640f (NIST, Gaithersburg, MD, USA), so that the NaCl powder and sample surfaces coincided with and were illuminated by the X-ray beam. Due to the design features of the desktop X-ray diffractometer used during measurements, the temperature in the sample chamber was kept at 313 ± 1 K. All XRD patterns were recorded in the range of diffraction angles 2*θ* from 6° to 141° with an angle step Δ2*θ*_step_ = 0.02° using the symmetric scanning mode *θ*-2*θ*.

The analysis of XRD patterns was carried out using the same methods as described earlier, for example, in [36]. These methods include use the program *EVA* [37] for determination of the XRD reflection parameters and for X-ray phase analysis using the Powder Diffraction File-2 database (PDF-2) [38], calculation of unit cell parameters from the corrected Bragg angle values 2*θ*_B_ and Miller indices *hkl* of the reflections using program *Celsiz* [39], and determination of the microstructural parameters utilizing WHP [40] and SSP [41] techniques encoded in the program *SizeCr* [42], which takes into account the pseudo-Voigt (pV) type [43] of XRD reflections in the calculations. The microstructural parameters obtained from the XRD data are mean sizes *D* of areas of coherent X-ray scattering (crystallites) and absolute mean values of microstrains *ε_s_* in them, and mean sizes *D*_0_ of crystallites in model of zero-microstrain (*ε_s_* = 0). When calculating the WHP and SSP points, the coefficients *K*_strain_ = 4 and *K*_Scherrer_ = 0.94 of the Wilson–Stokes [44] and Scherrer [45] equations were used, respectively, which connect the corresponding contributions to FWHM with the values of *ε_s_* and *D*. Some other details of the analysis are given in the Supporting Materials.

In addition to the XRD technique operating in Bragg–Brentano geometry, XRD studies were performed using reciprocal space mapping, similar to the technique used to analyze high-energy electron diffraction data. For this, a Super Nova diffractometer (Agilent Technologies, Inc.; Santa Clara, CF, USA) operating in kappa geometry with a two-dimensional (2D) detector (Atlas S2 CCD) and an X-ray gun with a copper cathode (*λ* = 1.5418 Å) was used. Mapping (as in the case of RHEED) consisted in measuring a series of XRD patterns depending on the angle of rotation around the normal to the sample. When the samples were rotated around the normal with a step of 0.5°, a series of images (360 images in a series) were measured, from which 3D maps of the reciprocal space were obtained using the software.

The study of the static magnetic properties of BaFe_12_O_19_/Al_2_O_3_(0001) single-layer structures was carried out using magnetometric and magneto-optical methods. The magnetization curves were measured by means of vibrating sample magnetometry (VSM) method using a vibrating magnetometer (Lake Shore Cryotronics, Westerville, OH, USA) with the magnetic field oriented both along the normal and in the plane of the structure. The magnetic field *H* varied in the range from +20 to –20 kOe. Measurements were carried out in the temperature range *T* = 100–300 K. Magnetization values were obtained using the measured magnetic moments, film thickness, and area. The saturation magnetization *M*_s_ and coercive fields *H*_c_ were calculated from hysteresis loops. To do this, dependences linear in the magnetic field were extracted from the experimental hysteresis loops, which are observed after saturation and are due to the magnetization induced by the magnetic field in the substrate. These loops were used to define the saturation magnetization as the magnetization independent of the magnetic field in strong fields, and the coercive field as the magnetic field corresponding to zero magnetization.

The magnetic field dependences of the polar magneto-optical Kerr effect (PMOKE) were measured on a polarimetric setup at wavelength *λ* = 405 nm at almost normal incidence of linearly polarized light (angle of incidence~1°). The magnitude of the magnetic field varied in the range from +25 to –25 kOe. The orientation of the magnetic field *H* was normal to the surface. During slow scanning of the magnetic field, the rotation of the plane of polarization of the reflected light was measured. To increase the sensitivity of polarization plane rotation measurements, the polarization of the incident (or reflected) light was modulated at a frequency *f*~400 Hz by the amplitude *α*_max_~1° using a Faraday cell. The sensitivity of rotation measurements was δ*α*~1″.

The spectral and field dependencies of the PMOKE were measured in the photon energy range *E_ph_* = 1.5–4 eV at RT in magnetic fields up to *H* = ±15 kOe. A compact shielded electromagnet was used to magnetize the sample at a frequency of ~2 Hz. During measurements, the magnetic field was oriented normally to the sample surface. The linearly polarized light incident from the monochromator was S-polarized. The analyzer was oriented at 45° to the plane of polarization of the incident light. The PMOKE value was calculated from the reflected light intensities for positive and negative magnetic fields *H*. The angle of incidence of light on the structure was *θ* = 52°. For getting spectral dependence of PMOKE at constant magnetic field *H* the intensity of reflected light was measured for +*H* and –*H* magnetic fields at each wavelength. For getting magnetic field dependence of PMOKE, the measurements were carried out for different magnitudes of magnetic field at constant wavelength *λ.* In addition, the spectral dependencies of the reflection coefficient of the samples were measured.

Spectral dependencies of transversal magneto-optical Kerr effect (TKE) were measured for in-plane orientation of magnetic field *H* oriented perpendicular to the light incidence plane. The magnitude of TKE = Δ*I*/*I*_0_ is a ratio between difference in reflected light intensities for positive +*H* and negative –*H* magnetic field Δ*I* = *I*(+*H*) − *I*(−*H*), and reflected light intensity *I*_0_ in demagnetized state.

Magnetorefractive effect (MRE) *MRE*^S^ was measured in geometry of TKE but for S-polarization of incident light. The magnitude of *MRE*^S^(*H*) = Δ*I*^MRE^/*I*_0_ where Δ*I*^MRE^ = *I*(0) − *I*(*H*). FMR was studied in thin (thickness *h* = 50 nm) films of hexaferrite BaFe_12_O_19_. For measurements, a JEOL-PE3 electron paramagnetic resonance radiospectrometer (JEOL Ltd., Tokyo, Japan) with a magnet providing a magnetic field *H* up to 1.3 T was used. Microwaves were generated using a Γ4-141 backward wave lamp generator operating in the frequency range of *f* = 38–53 GHz. FMR studies were carried out at a frequency *f* = 50 GHz. The recording system of the radiospectrometer was used to register the FMR signal. Magnetic field modulation with amplitude δ*H* = 5 Oe and frequency *f* = 100 kHz were used to increase the sensitivity of measurements.

## 3. Results and Discussion

### 3.1. Chemical Composition

**Figure 1:**
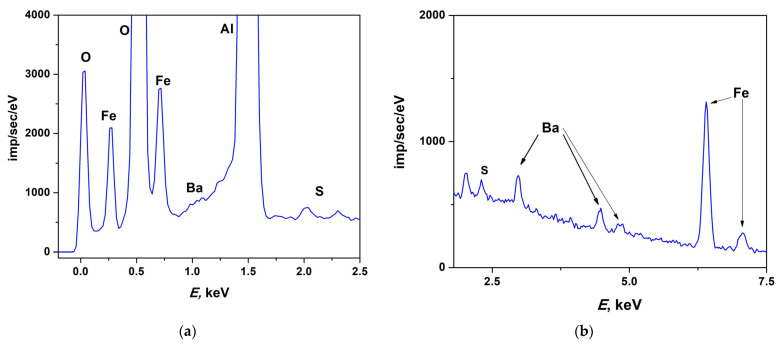
Typical EDX spectra of BaM structures measured by SEM for energy regions (**a**) 0–2.5 keV and (**b**) 2.5–7.5 keV.

EDX spectra were measured from both as-grown and annealed films. Typical EDX spectrum measured by means of SEM is presented in Figure 1. Table 2 shows the relative concentrations of Fe and Ba atoms in the as-grown and annealed films calculated from measured EDX spectra. In both cases, the Fe:Ba atomic ratio averaged over four measured points is close to the ideal composition (12:1), in particular (11.7:1) in the as-grown structure and (10.4:1) in the annealed one.

**Figure 2:**
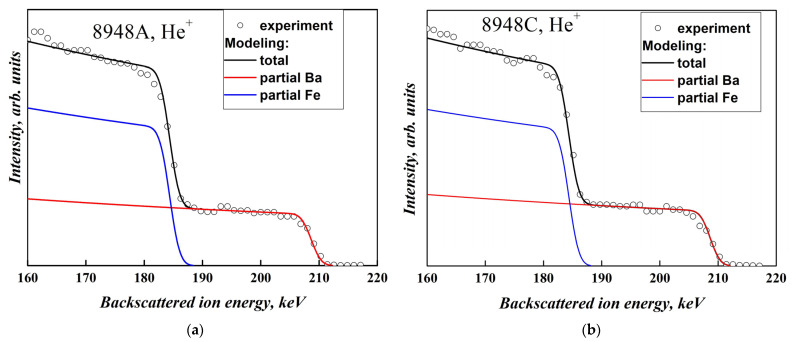
Experimental (circles) and model (solid lines) MEIS spectra measured using He^+^ ions for films with thickness *h* = 50 nm grown at 750 °C, (**a**) before (film #8948A) and (**b**) after annealing at 1000 °C for 60 min (film #8948C). Red and blue lines show contributions of Ba to Fe ions correspondingly in total signal, black line shows the modeling of total signal.

More accurate results were obtained using the MEIS technique. Figure 2 shows the experimental MEIS spectra measured with use of He^+^ ions and model spectra of the unannealed (#8948A) and annealed (#8948C) samples.

Model MEIS spectra were calculated using our original code, utilizing the basic principles of Rutherford Backscattering Spectrometry. The stopping cross sections for protons and helium ions were taken from [46,47,48]. The simulation showed that the Fe:Ba ratio is equal to 11.0 and 10.2 for the as-grown (#8948A) and annealed (#8948C) sample, respectively, which correlates with the data obtained using SEM. As can be seen from Figure 2, the decrease in the Fe:Ba ratio after annealing is mainly associated with a decrease in the Fe concentration, and the concentration of Ba ions changes slightly.

**Figure 3:**
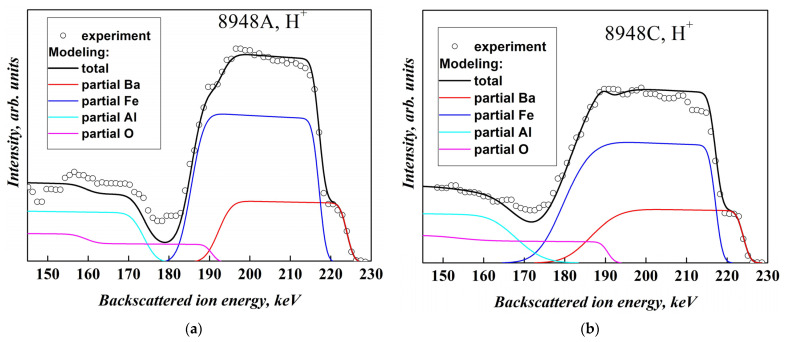
Experimental (circles) and model (solid lines) MEIS spectra measured using H^+^ ions for films with thickness *h* = 50 nm grown at 750 °C, (**a**) before (film #8948A) and (**b**) after annealing at 1000 °C for 60 min (film #8948C). Red, blue, cyan, and magenta lines in (**a**,**b**) show contributions to total signal of Ba, Fe, Al, and O ions, correspondingly, and the black line represents total calculated spectrum.

To measure the film thickness and its variations over area, as well as to estimate the oxygen content, MEIS experiments with H^+^ ions were carried out (Figure 3).

Simulation of the spectra presented in Figure 3 shows that the lateral density *N* of Ba*_x_*Fe*_y_*O*_z_* molecules with *x* + *y* + *z* = 1 (i.e., the total number of Ba, Fe, O atoms per 1 cm^2^ of the film) is *N* = 470 · 10^15^ molecules/cm^2^ and *N* = 580 · 10^15^ molecules/cm^2^ in the as-grown and annealed films, respectively. An estimate of the film thickness *h* based on the assumption that the mass density of the films corresponds to the density of BaFe_12_O_19_ crystals (i.e., *ρ* = 5.01 g/cm^3^) gives values *h* = 54 nm and 67 nm for the samples #8948A and #8948C, correspondingly, which is close to the RHEED results (*h* = 50 nm).

It is important to note that, in the as-grown film, the value of *N* does not depend on the in-plane coordinates, i.e., the film is laterally homogeneous. In contrast, in the annealed film, this parameter is characterized by a remarkable dispersion of 80·10^15^ atoms/cm^2^. In fact, the shape of the low-energy Ba + Fe signal front is rather sharp for as-grown sample (180–195 keV, Figure 3a) and flat in the annealed film (170–195 keV, Figure 3b).

An indirect estimate of the oxygen content was obtained by comparing the intensity of signal from the film (right sides in Figure 3a,b) and the substrate signal (left side). Such an estimate showed the atomic ratio O:Ba~(14:1) and ~(19:1) for the as-grown and annealed 8948 film, respectively.

Thus, both approaches, EDX and MEIS, show that the atomic ratios in the as-grown and annealed films are close to those expected in BaFe_12_O_19_. This indicates that the number of ions on the substrate surface after deposition is sufficient for the nucleation and crystallization of BaM hexaferrite. The Fe:Ba atomic ratio in the annealed sample is slightly lower than in the as-grown one, which indicates that the film composition is depleted of iron upon annealing at high temperatures. After annealing, both the layer thickness and their dispersion increase, which may be due to BaFe_12_O_19_ crystallization during annealing. At the same time, as a result of annealing, the O:Ba atomic ratio approaches the ideal one.

### 3.2. Surface Morphology

**Figure 4:**
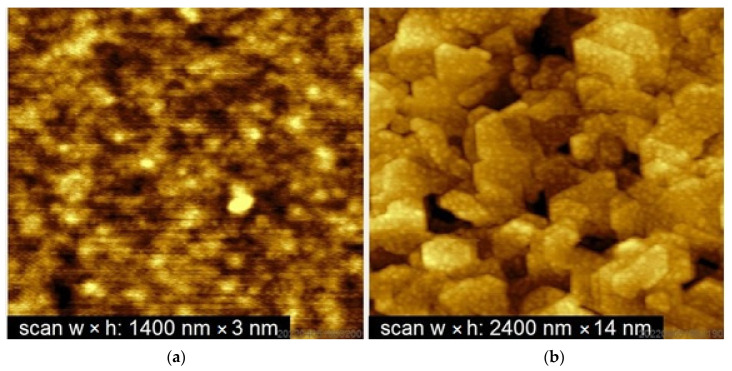
AFM images of the surface of BaM hexaferrite layers (*h* = 300 nm) grown at 750 °C (**a**) before (film #8954A) and (**b**) after annealing at 1000 °C for 60 min (film #8954C).

Figure 4 shows the AFM images of structures #8954A (as-grown, *h* = 300 nm) and #8954C (annealed in duration of 60 min, *h* = 300 nm). The unannealed film #8954A demonstrates a rather smooth surface with no evidence of the presence of nanocrystallites (Figure 4a). The average roughness of such a surface is a root mean square (RMS) value of ~2 nm over an area of 2 × 2 μm^2^. In contrast, the surface of the annealed sample #8954C (Figure 4b) consists of a set of nanocrystallites with a pronounced faceting, which is typical for crystals with a 6th-order axis directed approximately normal to the substrate. The structure contains nanocrystals in the form of regular hexagons, hexagons with different side lengths, and triangles. Note that the orientations of the sides of the nanocrystallite faces located at different edges of the pattern are practically parallel to each other, which indicates a correlation of crystallographic directions in them.

Thus, studies of the surface morphology using AFM showed that the surface of the annealed films is composed of closely packed nanocrystallites in which a 6th-order axis is oriented approximately normal to the surface, which confirms the presence of BaM hexaferrite. In contrast, the presence of nanocrystallites is not observed in the AFM images of unannealed structures.

### 3.3. RHEED Study of Crystal Structure

**Figure 5:**
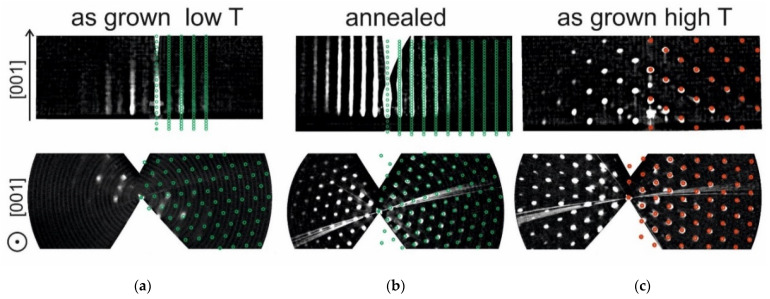
Three-dimensional reconstruction of RHEED data for (**a**) film #8954A grown at 750 °C (*h* = 300 nm) without annealing, (**b**) #8954B (*h* = 300 nm) grown at 750 °C and annealed for 60 min at 1000 °C, and (**c**) #8947 grown at 1000 °C without annealing (*h* = 50 nm). The green and red circles represent the nodes of the model lattice calculated using the parameters of the BaM hexaferrite bulk crystal.

Figure 5 presents reciprocal space images obtained by 3D mapping of RHEED patterns in films #8954A (*T*_gr_ = 750 °C, as-grown), 8954B (*T*_gr_ = 750 °C, annealing at *T*_ann_ = 1000 °C for 60 min), and #8947 (*T*_gr_ = 1000 °C, as-grown).

The RHEED patterns of hexaferrite layers grown at temperatures of 700–850 °C (not annealed, see Table 1) more or less correspond to the bulk structure of BaM hexaferrite (Figure 5a). However, the increased width of the reflections and the uneven distribution of the intensity of the reflections in these patterns indicate a relatively low crystalline quality of the grown films. It is also seen that every second rod has a significantly higher intensity, which indicates a strong violation of the long-range order. In contrast, films grown at 700–850 °C and then annealed in air at 1000 °C, show RHEED images ideally modeled by the BaM bulk lattice (Figure 5b). It can be seen that the number of reflections and the signal-to-background ratio are much better compared to the unannealed samples. All rods have the same intensity, which indicates the presence of long-range order. Films grown at temperatures *T*_gr_ = 900–1000 °C differ significantly from samples grown at lower temperatures. The RHEED patterns of such samples are well modeled by the α-Fe_2_O_3_ (hematite) lattice (Figure 5c). The formation of α-Fe_2_O_3_ is apparently associated with repeated sputtering (re-evaporation caused by elevated temperature) of Ba ions at high temperatures. Accounting for the re-sputtering process can explain the effect of annealing at 1000 °C on the crystal quality of films grown at low temperatures. In films grown at *T*_gr_ = 700–850 °C, the re-sputtering is small and the Ba ions are distributed more or less uniformly inside the hexaferrite film. Annealing, on the other hand, leads to a redistribution of Ba positions and the occupation of Ba ions in places corresponding to the BaM crystal structure, which leads to the formation of a high-quality BaM layer with improved magnetic properties (see below). Film growth at 1000 °C is accompanied by the disappearance of Ba ions from the film and the formation of the α-Fe_2_O_3_ structure.

Thus, the results of studying the crystal structure using RHEED confirm the conclusions of the previous section devoted to the study of surface morphology using AFM. In the unannealed films, the RHEED patterns do not show the presence of a well-defined BaM hexaferrite structure with long-range order. They may contain uncorrelated small nuclei. A clear picture of RHEED, which fully corresponds to the presence of the hexaferrite structure, appears only in annealed films.

### 3.4. XRD Studies

**Figure 6:**
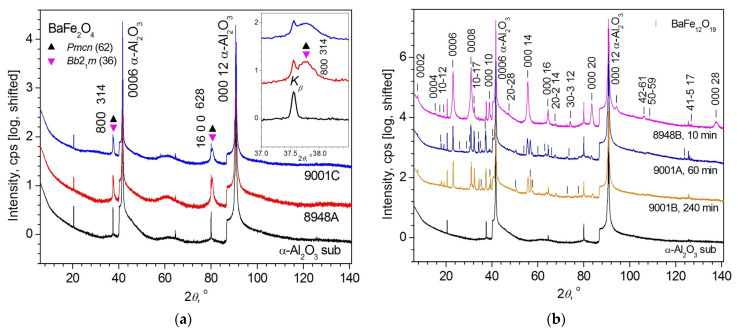
XRD patterns (*θ*-2*θ* scans) of the samples #9001 and #8948, and α-Al_2_O_3_(0001) substrate (**a**) before annealing, and (**b**) after annealing (see Table 1). The duration of annealing of each sample is indicated. On panel (**a**), different triangular symbols show the angular positions of the observed reflections of various possible modifications of BaFe_2_O_4_ (space group (sp. gr.) *Bb*2_1_*m* (36) and sp. gr. *Pmcn* (62)) according to the unit cell parameters from the PDF-2 database, entries 00-046-0113 and 01-077-2337, respectively. The inset in (**a**) shows part of the XRD patterns of the films before annealing in vicinity of the BaFe_2_O_4_ reflection with Miller indices *hkl* = 800 for modification with sp. gr. *Bb*2_1_*m* (36) (or *hkl* = 314 for phase with sp. gr. *Pmcn* (62)) on an enlarged scale. In panel (**b**), the observed reflections of BaFe_12_O_19_ (sp. gr. *P*6_3_/*mmc* (194) according to PDF-2 entry 00-027-1029) are marked with bar symbols at the positions of the Bragg angles 2*θ*_B_^calc^ obtained using the BaFe_12_O_19_ unit cell parameters calculated in this work. The Miller–Bravais *hkil* indices of some selected BaFe_12_O_19_ reflections are indicated. For better visualization, the XRD patterns in (**a**,**b**) are shifted vertically.

The results of XRD measurements of the film samples are shown in Figure 6.

The obtained interplanar distances in unannealed samples and the calculated parameters of unit cells in annealed samples are given in Table 3 and Table 4. The results of determining the parameters of the microstructure from the analysis of the observed pseudo-Voigt (pV) profiles (0.636 < *FWHM*/*B*_int_ < 0.939 [39]) of XRD reflections using the Williamson–Hall plot (WHP) and the Size–Strain plot (SSP) are presented in the same Table 3 and Table 4. The WHP and SSP graphs are given in Figures S1 and S2 of Supplementary Materials.

Estimated standard deviations (e.s.d.s., see the note in the Supplementary Materials) of the structural and microstructural parameters are shown in Table 3 and Table 4 in round brackets. As is seen, the *D* and *ε*_s_ values obtained by WHP and SSP techniques are close in the limits of e.s.d. for the samples without annealing (Table 3) and for the sample #8948D after annealing (Table 4). For the sample #9001C after different annealing durations, there are significant differences (Table 4). However, high values of the *R*_cod_ coefficient (*R*_cod_ = 71.95–95.22%) in the case of SSP analysis compared with significantly lower values of *R*_cod_ = 2.03–15.06% for WHP indicate more accurate values of microstructural parameters obtained using SSP, which were used for further analysis.

In the unannealed films, only two reflections from the film are present in XRD patterns. These reflections are well-identified as reflections of two possible orthorhombic modifications of BaFe_2_O_4_ (Figure 6a). Both reflections are reflections of a different order from the same type of parallel diffracting atomic planes. No reflections from a set of planes of another type are observed, which indicates a strong predominant orientation of the unannealed films. Judging by the Miller indices of the reflections, there is a preferential orientation along the [100] or [314] direction depending on the possible modification of BaFe_2_O_4_. Due to the small number of XRD reflections, measurements do not allow one to choose between these two modifications of BaFe_2_O_4_.

All observed non-substrate reflections of annealed films 8948B, 9001A, and 9001B grown at *T*_gr_ = 700–750 °C (Table 1) are attributed to the BaFe_12_O_19_ crystalline phase (sp. gr. *P*6_3_/*mmc* (194)). There are no reflections of other crystalline phases, including reflections of hematite (α-Fe_2_O_3_, sp. gr. $R\overset{-}{3}c$ (167)), which are formed in films grown at higher *T*_gr_ = 900–1000 °C (see Section 3.3 and Figure S3 of Supporting Materials). Thus, annealing leads to crystallization in all studied films of the BaFe_12_O_19_ compound of hexagonal syngony with a predominant orientation along the [*hkil*] = [0001] direction (Figure 6b), as evidenced by the presence of a large set of different orders of the 000*l* reflection with increased intensities. However, the predominant orientation is not as strong as in unannealed films, since XRD patterns also contain not only 000*l* reflections, but also *hkil* reflections.

**Figure 7:**
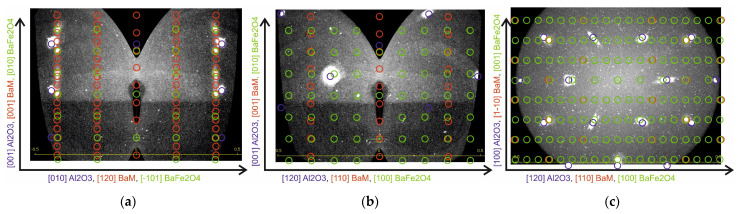
Cross sections of reciprocal space maps from an unannealed sample 300 nm thick in different planes. Blue, red, and green circles in (**a**–**c**) correspond to calculated pattern of Al_2_O_3_, BaM, and BaFe_2_O_4_, respectively.

**Figure 8:**
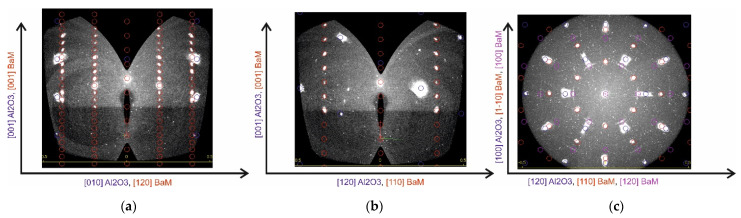
Cross sections of reciprocal space maps from an annealed sample 300 nm thick in various planes. Blue, red, and violet circles in (**a**–**c**) correspond to Al_2_O_3_, dominant phase of BaM, and textured phase of BaM, respectively.

Thus, XRD studies showed that only the orthorhombic BaFe_2_O_4_ phase, which consists of highly stressed nanocrystallites (*ε*_s_ = 0.159(8)–0.22(3)%, Table 4), manifests itself in unannealed samples. After annealing, the BaFe_2_O_4_ phase disappears in all structures and the hexagonal BaFe_12_O_19_ phase is present. In a #8948B structure (film thickness *h* = 50 nm), a 10 min annealing virtually eliminates microstrains (*ε*_s_ = 0.03(36)% in comparison to *ε*_s_ = 0.159(8)% in the unannealed sample). In thick films 9001A and 9001B (*h* = 500 nm), annealing for 60 to 240 min does not result in peeling, but reduces the amount of microstrain from *ε*_s_ = 0.22(3)% to 0.17(1)–0.18(2)% (Table 4). The results of XRD studies of the films using reciprocal space mapping are shown in Figure 7 and Figure 8.

Figure 7 shows that the substrate reflections are well modeled by the reciprocal lattice of a bulk sapphire crystal. In contrast, the film reflections in none of the azimuths correspond to the BaM model reciprocal lattice (red circles in Figure 7a,b). In the direction normal to the sample, the interplanar spacing coincides with good accuracy with the interplanar spacing in BaFe_2_O_4_ (green circles in Figure 7a,b). However, in the plane of the sample (Figure 7c), apparently, there is a large disorder.

For the annealed sample, XRD mapping data (Figure 8a–c) show good agreement between the model reciprocal lattice of BaM and the observed reflections. It can be concluded that the dominant lattice of the BaM film is rotated by 30° relative to the sapphire lattice in the sample plane. However, it can be seen that in the section constructed in the plane of the sample (Figure 8c), in addition to the dominant BaM lattice (red circles), there is one more phase with interplanar spacings corresponding to the reciprocal BaM lattice. This phase does not have a 30° turn and tends to be textured (has a fairly large, on the order of several degrees, spread in rotation angles around the normal to the sample surface), since the reflections from this phase have the shape of sphere sectors. According to the intensity ratios, it can be said that the volume of the textured phase is much smaller than the volume of the BaM dominant phase. Note that some of the reflections present in the data obtained when measuring in reflection geometry (*θ*-2*θ* scans, Figure 6b) using a linear (1D) detector were not reliably recorded in the course of 3D mapping; this may be due to the lower sensitivity of the 2D detector used in mapping (on the specular reflection curves, the data reflections have an intensity several orders of magnitude lower than the main BaM phase). Apparently, the XRD reflections found in the reflection geometry are related to the textured phase found using XRD mapping. It can be hoped that with further optimization of the annealing conditions, it will be possible to get rid of the textured BaM phase, which, in our opinion, should lead to an improvement in the magnetic characteristics of the BaM films.

In conclusion of this section, let us consider the results obtained from the point of view of understanding the processes occurring during growth. Film growth at the growth temperature *T_gr_* = 1000 °C leads to the appearance on the substrate mainly of hematite α-Fe_2_O_3_ phase. Obviously, this is due to the strong re-evaporation of Ba ions from the film at such a high growth temperature, which excludes the appearance of hexaferrite. A decrease in the growth temperature to *T_gr_* = 700–750 °C is no longer accompanied by a strong evaporation of Ba ions, and the amount of Ba, Fe, and O ions on the substrate required to obtain BaM hexaferrite turns out to be quite sufficient, both in annealed and unannealed structures. However, immediately after film growth, it is not hexaferrite that is formed on the substrate, but rather highly strained BaFe_2_O_4_ nanocrystallites with a spinel structure. This happens, on the one hand, because the growth process by the LMBE method is a very nonequilibrium process, in which a large amount of material evaporated from the target falls on the substrate in a very short time, which should eventually form a hexaferrite structure, and on the other hand, due to the complex structure of hexaferrite, the unit cell of which consists of a certain sequence of spinel and hexagonal blocks. It is the spinel block BaFe_2_O_4_ that is one of the “bricks” of the hexaferrite lattice. It seems that the formation of “bricks” at the first stage is required for the construction of hexaferrite, and their ordering into the structure of hexaferrite at the second stage. Perhaps, in the films obtained immediately after growth, we observe the result of only the first stage. The implementation of the second stage requires annealing at *T_ann_* = 1000 °C, which is higher than the growth temperature. Despite the high annealing temperature, evaporation of Ba ions from the film, at least for a short annealing time of 10 min, does not occur, since they are already embedded in the Ba_2_FeO_4_ spinel blocks.

Note that annealing must be carried out in air, i.e., at high oxygen concentrations. Experiments have shown that annealing at *T_ann_* = 1000 °C directly in the growth chamber, at a low oxygen pressure of up to 0.2 mbar, does not lead to the formation of a hexaferrite structure. Apparently, this indicates that, during annealing, a larger amount of oxygen must be introduced into the film from the outside.

As a result, the formed crystalline hexaferrite phase in thin 50 nm films (#8948B), annealed in a short time of 10 min, is characterized by the nanocrystallite sizes *D*~40 nm comparable with the film thickness, absence of microstrains in them and the unit cell parameters that are closest to the PDF-2 tabular values (Table 4).

Annealing in air at *T_ann_* = 1000 °C of thick 500 nm-films at long durations of 60 min (#9001A) and 240 min (#9001B) leads to formation of larger crystallites with noticeable microstrains (according to SSP analysis, *D* are larger than ~100 nm, *ε*_s_~0.1%). In addition, an increase in the annealing duration leads to a sequential decrease in the values of the unit cell parameters. This is probably due to the fact that during long-term annealing, nevertheless, some of the Ba atoms can still evaporate (with the concomitant evaporation of oxygen or a change in the Fe valence to maintain electrical neutrality), but with the preservation of the hexaferrite structure. As a result of a larger difference from the tabular values of the lattice parameters, microstrains appear in the emerging hexaferrite nanocrystallites.

### 3.5. Static Magnetic Properties

#### 3.5.1. PMOKE Measurements

**Figure 9:**
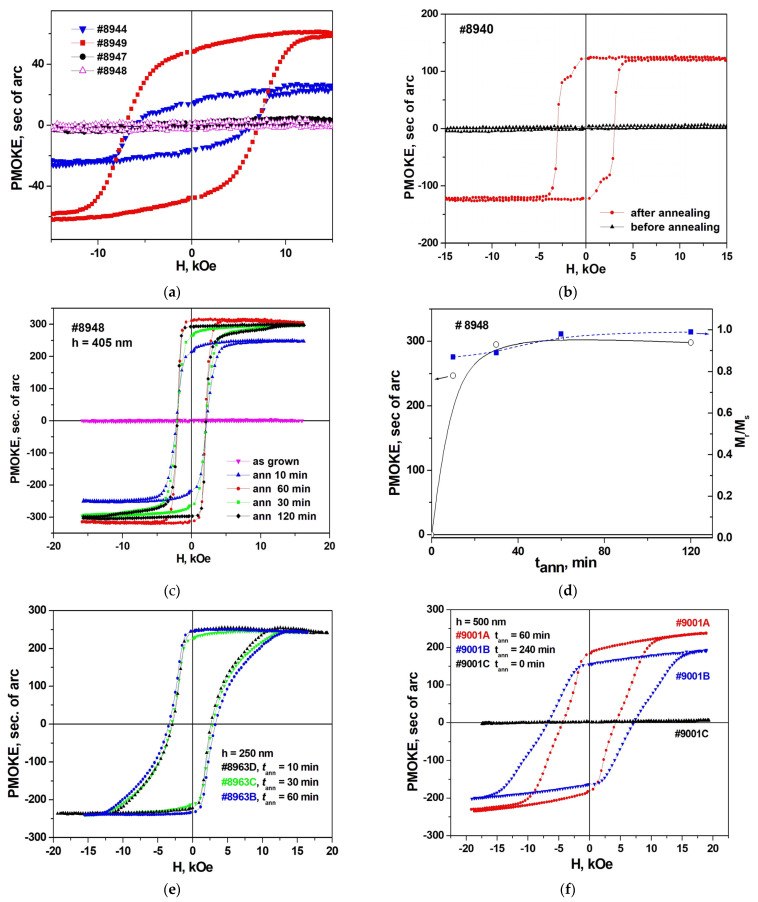
PMOKE magnetization curves of unannealed samples (**a**) #8944, #8949, #8947, #8948, and (**b**) sample #8940 before and after annealing, and (**c**) PMOKE magnetization curves of the sample #8948 (*T*_gr_ = 750 °C, film thickness *h* = 50 nm) versus annealing time (*t*_ann_ = 0 (i.e, as grown), 10 min, 30 min, 60 min, and 120 min) at temperature *T*_ann_ = 1000 °C. (**d**) Annealing time dependence of the PMOKE value in saturation (circles) and the ratio of remanence to saturation magnetization *M*_r_/*M*_s_ (blue squares). PMOKE magnetization curves versus annealing time (**e**) of the sample #8963 (*h* = 250 nm, annealing time *t*_ann_ = 10 min, 30 min, and 60 min) and (**f**) of the sample #9001 (*h* = 500 nm, *t*_ann_ = 60 min and 240 min).

The study of magnetic hysteresis loops using PMOKE polarimetric technique showed a significant difference between unannealed and annealed structures. Most of the as-grown structures do not show the presence of any magnetic moment. Only in a few structures were observed weak and diffuse loops, the shape of which is far from rectangular (Figure 9a).

In contrast, in all as-grown films, even in those that did not show magnetic properties, annealing led to the appearance of pronounced and almost rectangular magnetic hysteresis loops (Figure 9b). The effect of annealing on the shape of hysteresis loops in films #8948 (*h* = 50 nm), #8963 (*h* = 250 nm), and #9001C (*h* = 500 nm) is shown in Figure 9c,e,f. The shape of the hysteresis loops of the annealed structures is rather close to rectangular (Figure 9b,c). It is important that the remanent PMOKE value is very close to the saturation one. This indicates that after saturation of the film in a strong field, the direction of the residual magnetization in zero field remains practically normal to the plane of the film. Figure 9d shows the value of PMOKE in saturation (proportional to *M*_s_) and relative remanence *M*_r_/*M*_s_ versus annealing time *t*_ann_ in structure #8948 (*h* = 50 nm). With an increase in *t*_ann_ to 120 min, the value of *M*_s_ first increases sharply, and in the range *t*_ann_~40–120 min it changes very weakly. The relative residual magnetization *M*_r_/*M*_s_ in the range of 10–120 min slightly increases from 0.9 to 1.

It is important to note that the shape of the hysteresis loops in the annealed structures essentially depends on the thickness *h* of the hexaferrite layer. Figure 9e,f show hysteresis loops in films #8963 (*h* = 250 nm) and #9001 (*h* = 500 nm) with different annealing time. The shape of the loops in these structures is far from being rectangular, due to the tightening of the branches at |**H**| > |**H**_c_| in sample #8963 and with a strong slope of the branches in sample #9001. Note that an increase in the annealing time in such a structure leads to “deterioration” of the loops, i.e., to an increase in *H*_c_ and an even greater difference in the shape of the loop from a rectangular one.

Thus, studies of hysteresis loops using PMOKE have shown that only annealed structures demonstrate magnetic properties. They exhibit loops with a large remanence magnetization *M*_r_/*M*_s_, which is required for microwave devices based on direct bulk spin waves. The narrowest and most rectangular loops appear in samples with thin (*h* = 50 nm) hexaferrite layers. In structures with layer thickness *h* = 250–500 nm, the loop rectangularity decreases with increasing *h*. An increase in the annealing time in such structures leads to an even greater deviation of the loop shape from a rectangular shape.

#### 3.5.2. VSM Measurements

**Figure 10:**
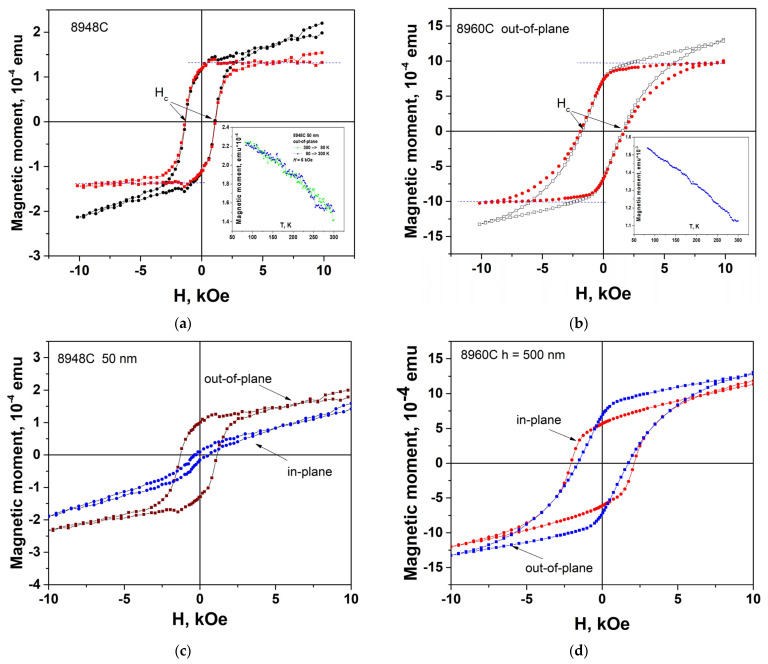
Magnetization curves measured with VSM of the samples (**a**,**c**) #8948C (*h* = 50 nm, *t*_ann_ = 60 min) and (**b**,**d**) #8960C (*h* = 500 nm, *t*_ann_ = 120 min). The VSM measurements for films (**a**) #8948C and (**b**) #8960C are carried out in magnetic field *H* normal to the film surface. The red dots in (**a**,**b**) are obtained after subtracting the linear dependence associated with the substrate. Coercive field *H*_c_ obtained after the subtraction of the linear dependence is indicated in (**a**,**b**). Insets in (**a**,**b**) show temperature dependence of magnetic moment. (**c**) Comparison of hysteresis loops for out-of-plane and in-plane orientation of the magnetic field in #8948C. (**d**) Comparison of hysteresis loops for out-of-plane and in-plane orientation of the magnetic field in structure #8960C.

The magnetization curves of annealed samples #8948C (*h* = 50 nm, *T*_ann_ = 1000 °C for *t*_ann_ = 60 min) and #8960C (*h* = 500 nm, *T*_ann_ = 1000 °C, *t*_ann_ = 120 min) measured by VSM are obtained for a magnetic field oriented normally (Figure 10b,d) and in the plane of the BaM layer (b,d). In contrast to the loops observed in PMOKE, the magnetic loops manifest themselves against the background of linear dependencies *M*(*H*) due to the substrate. This background does not manifest itself in PMOKE measurements, since light at a wavelength of 405 nm does not reach the substrate even at a film thickness *h* = 50 nm. Hysteresis loops of thin #8948C film for in-plane and out-of-plane orientations of the magnetic field are different, as expected. In sample #8960C with a film thickness *h* = 500 nm, a completely different picture is observed in comparison to the thin (*h* = 50 nm) sample #8948C (cf. Figure 10d,c).

For #8960, the hysteresis loop in the out-of-plane geometry (the same shape as the PMOKE loop) turns out to be comparable in size to the loop in the in-plane geometry (Figure 10d). This indicates that the film #8960 contains regions with a very wide distribution of the direction of the axis of easy magnetization, i.e., the film is very inhomogeneous. This correlates with the conclusions of the previous Section 3.5.1 devoted to PMOKE studies, according to which thin films (*h* = 50 nm) of BaM hexaferrite after annealing turn out to be much more uniform than thick films (*h* = 250–500 nm).

BaM hexaferrite films on Al_2_O_3_(0001) substrates should have uniaxial anisotropy with an easy magnetization axis normal to the surface, so the magnetization switching in out-of-plane geometry should occur by domain wall nucleation and motion, and in in-plane geometry by magnetization rotation. The appearance of a weak loop in the *M*(*H*) dependence in the in-plane geometry in thin film #8948C (Figure 10c) indicates that the film also is not ideal due to presence in it of regions with the direction of the easy axis somewhat different from the normal one.

The appearance of irreversible behavior of the magnetization in in-plane geometry in BaM hexaferrite films was associated with the existence of an interdiffusion layer caused by mutual diffusion of substrate and film ions at the interface [49]. Such diffusion can lead to the appearance of regions with slightly different orientations of the easy axis and, as a result, magnetic hysteresis loops in such a geometry. One of the arguments in favor of this mechanism was a considerable increase in the hysteresis loop, observed in a film, grown on an Al_2_O_3_ substrate by laser deposition in in-plane geometry after the sample was annealed at a temperature of 900 °C for 60 min.

In our samples with thin layers of BaM hexaferrite (*h* = 50 nm), the in-plane magnetic hysteresis is small. In thick (*h* = 250–500 nm) hexaferrite films strong hysteresis loops are observed in this geometry. In general, this corresponds to the possible manifestation of an interdiffusion layer, since the growth time of thick films is longer than that of thin ones. However, judging by the behavior of hysteresis loops in out-of-plane geometry, annealing affects thin and thick films differently. In thin films, this leads to an increase in the *M*_r_/*M*_s_ ratio with increasing annealing time (Figure 9d), i.e., to a greater rectangularity of the loop. In thick films, on the contrary, annealing leads to a deterioration in the shape of the loop and an increase in *H*_c_. This indicates the manifestation of more complex loop formation mechanisms, at least in thin films.

The magnetization of the structures was calculated from the experimentally measured value of magnetic moment, using the known layer thickness *h*, and the sample area *S*. In film #8948C (*h* = 50 nm, *t*_ann_ = 60 min), the saturation magnetization is *M*_s_ = 340 emu/cm^3^ (4π*M*_s_ = 4.3 kG), which is in good agreement with the value for a bulk crystal. The magnetization value for thick film is much lower *M*_s_ = 230 emu/cm^3^ (4π*M*_s_ = 2.9 kG). A decrease in measurement temperature *T* leads to an increase in magnetization *M*_s_ in both thin and thick films (see insets in Figure 10a,b).

Thus, studies of the magnetic moment using VSM showed that the magnetization of a samples with a thin film of hexaferrite BaM (*h* = 50 nm) depends significantly on the orientation of the magnetic field relative to the plane of the structure, in accordance with what is observed in bulk samples. In contrast, in the sample with a thick hexaferrite layer (*h* = 500 nm), this difference turns out to be insignificant, which indicates a strong spread in the orientation of the easy magnetization axis in this structure. Also important is the difference in the magnitude of magnetization in structures with thin (*h* = 50 nm) and thick (*h* = 500 nm) BaM hexaferrite films. The low values of magnetization in a film with a thickness of *h* = 500 nm indicate an inhomogeneous structure of thick films.

### 3.6. Magnetooptical Spectroscopy

**Figure 11:**
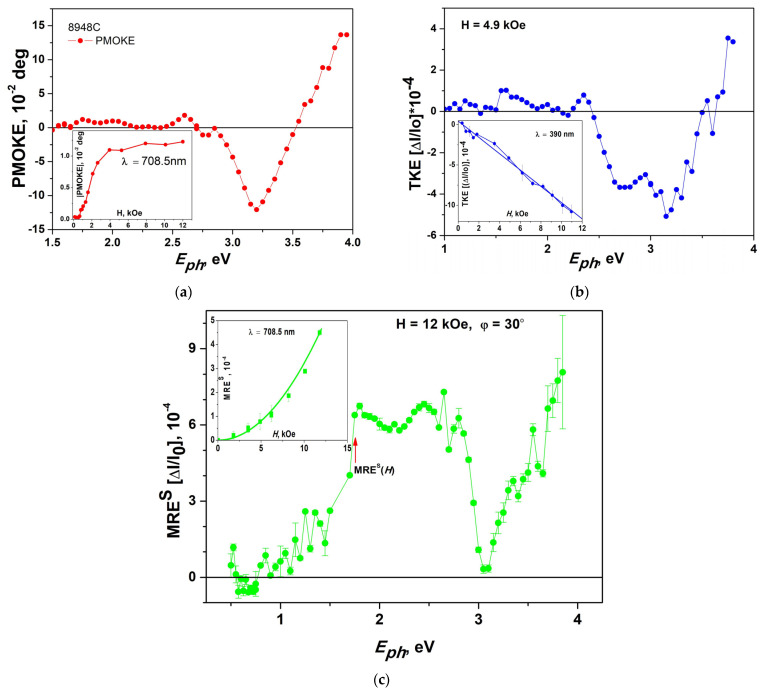
Magneto-optical spectra of #8948C sample. (**a**) PMOKE spectrum measured with out-of-plane magnetic field *H* = 4 kOe. Inset shows magnetic field dependence of PMOKE measured with spectroscopic setup. Note that the shape of magnetic field dependence is different from one measured with polarimetric setup (Figure 9) because in spectroscopic setup the magnitude of PMOKE(*H*) is obtained as a difference between signals measured for +*H* and −*H* magnetic fields. (**b**) TKE measured with in-plane magnetic field *H* = 4.9 kOe. Inset shows magnetic field dependence of TKE for *λ* = 390 nm. Red dashed line in the inset corresponds to fitting of the experimental points, linear with respect to *H*. (**c**) MRE^S^ spectrum. Inset shows magnetic field dependence of MRE^S^ for *λ* = 708 nm. Red dashed line in the inset corresponds to fitting of the experimental points, quadratic with respect to *H*.

The spectral dependencies of PMOKE and transverse Kerr effect (TKE) measured for the #8948C (*T*_gr_ = 750 °C, *h* = 50 nm) sample are shown in Figure 11a. As is known [31,50,51], the measured rotation of the light polarization plane in BaM hexaferrite films in the region of photon energies *E*_ph_ > 2.6 eV is related to the polar Kerr effect only. At lower photon energies, the Faraday effect adds to the rotation as the film becomes more transparent. In this photon energy region, the position of the bands in the PMOKE spectrum and their intensity depend on the ratio between the light wavelength and the film thickness [49]. A characteristic feature of the PMOKE spectrum is the appearance of strong bands of different signs in the region of *E*_ph_~3.18 eV (PMOKE ≈ −0.15 deg) and *E*_ph_~4.6 eV (PMOKE~0.28 deg) [49]. Similar bands were also observed in BaM hexaferrite films, grown by the metallo-organic decomposition method [37], at *E*_ph_~3.15 eV and 4.25 eV, as well as in sputtered PbFe_12_O_19_ [52] and SrFe_12_O_19_ [53]. The nature of these bands is associated with a charge transfer (from Fe^3+^ to O^2-^) optical transitions for Fe^3+^ ions in octahedral and tetrahedral positions [49]. Figure 11 clearly shows manifestation of PMOKE band at *E*_ph_~3.2 eV (PMOKE = −0.12 deg), a change of the PMOKE sign at *E*_ph_~3.6 eV, and an increase of PMOKE positive values above *E*_ph_~3.6 eV.

The TKE spectrum measured for *H* = 4 kOe (Figure 11b) also shows the band centered at *E*_ph_~3.2 eV. Note that because magnitude of TKE is proportional to in-plane magnetization component, which saturates at *H* = *H*_a_~15 kOe, the values of TKE measured for *H* = 4.9 kOe are smaller than when all magnetization is oriented in the plane of the film (see inset in Figure 11b).

The optical band at *E*_ph_ ≈ 3.2 eV manifests itself also in the spectrum of magneto-reflection effect MRE^S^, which is measured in geometry of TKE, but for the S-polarization of incident light (Figure 11c). MRE^S^ quadratically depends on magnetic field *H* (see inset in Figure 11c), in contrast to TKE, which reveals a linear dependence on the magnetic field *H* (see inset in Figure 11b). This shows that MRE^S^ is proportional to the square of in-plane magnetization component caused by applied in-plane magnetic field *H*, in contrast to TKE which is linear in this component. The reflectivity quadratic in magnetization components was discovered in 1969 [54] and named the orientational magneto-optical effect. Later, it was studied in Fe–Ni, Fe–Ti, Fe–V alloys [55], orthoferrites, orthochromites, and orthomanganides [56].

To conclude this subsection, we note that the studies of magneto-optical spectroscopy show that both the PMOKE and TKE spectra and the MRE spectra confirm the presence of BaM hexaferrite on the (0001) Al_2_O_3_ substrate in the studied samples.

### 3.7. Magnetization Dynamics

**Figure 12:**
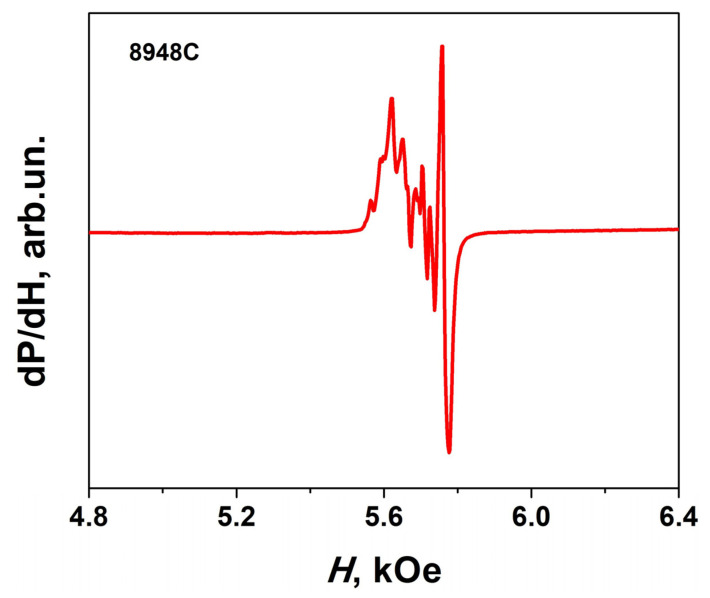
FMR spectrum in annealed #8948C sample (*h* = 50 nm, *T_ann_ =* 1000 °C, *t_ann_* = 60 min).

The FMR spectrum at a frequency *F* = 50 GHz with a magnetic field direction normal to the film plane for the annealed sample #8948C (*h* = 50 nm, *T*_ann_ = 1000 °C, *t*_ann_ = 60 min) is shown in Figure 12. The FMR band consists of a set of narrow FMR lines with resonant fields in the range *H*_res_ = 5.6 kOe–5.8 kOe. Resonant field and width of the main FMR line are *H*_res_ = 5.76 kOe and Δ*H*_res_ = 20 Oe, respectively. The presence of a set of lines at lower fields is apparently associated with the magnetic inhomogeneity of the layer due to the spread in the anisotropy field, the direction of the easy axis, and so on. We note that we observed such a splitting of FMR lines earlier in YIG/GGG(111) structure [6]. Using the values of the resonant field for the main line *H*_res_ = 5.76 kOe, the value of the magnetization obtained using the VSM, 4π*M*_s_ = 4.3 kG, the measurement frequency *F* = 50 GHz, we obtained an estimate of the anisotropy field *H*_a_ = 16.4 kOe, which is quite close to the values *H*_a_ = 17.0 kOe for 4π*M*_s_ = 4.3 kG in the BaFe_10.5_Mn_1.5_O_19_/Al_2_O_3_(0001) structure [57].

## 4. Conclusions

The main result of these studies, carried out using various experimental techniques, consists in observation of almost rectangular hysteresis loops for out-of-plane orientation of magnetic field in heterostructures with a thin epitaxial layer of BaM hexaferrite grown by laser molecular beam epitaxy on Al_2_O_3_(0001) substrate. Such loops are observed in thin (*h*~50 nm) films grown at *T*_gr_ = 700 °C after a short (*t*_ann_~5–10 min) annealing in air at *T*_ann_ = 1000 °C.

It is important to note that the key role in the formation of the epitaxial structure of hexaferrite is played by the evaporation of Ba ions from the substrate during growth process. If BaM films are grown at a temperature *T*_gr_ = 1000 °C, then hematite α-Fe_2_O_3_ is mainly formed on the substrate, because of a strong evaporation of Ba ions from the substrate at this temperature. During film growth at *T*_gr_ = 700 °C, the amount of Ba, Fe, and O ions on the substrate is sufficient for the formation of BaFe_12_O_19_. Nevertheless, at a stage of “as-grown”, mainly BaFe_2_O_4_ nanocrystallites are formed on the Al_2_O_3_ surface. Upon subsequent annealing at *T*_gr_ = 1000 °C, BaFe_2_O_4_ recrystallizes into BaM hexaferrite. In this case, Ba ions do not evaporate from the film.

An important result is the fact that the structural and magnetic properties of the annealed films depend significantly on the film thickness. In thin (*h*~50 nm) annealed BaFe_12_O_19_ films, a hexaferrite crystal structure is realized with the direction of the hexagonal axis and the easy magnetization axis close to the normal to the plane of the structure. The magnitude of the magnetic moment *M* and the anisotropy magnetic field *H*_a_ in such films are close to those of bulk BaM samples, and relatively narrow FMR lines are observed in them. The magnitude of the magnetization and the anisotropy field is close to similar films known from the literature.

In contrast, thicker BaFe_12_O_19_ films (*h* = 250 nm–500 nm), also annealed at *T*_ann_ = 1000 °C, exhibit a structure with strong fluctuations in the direction of the easy magnetization axis, which leads to the appearance of hysteresis loops for in-plane the magnetic field comparable to the loops observed for out-of-plane one, as well as strong differences in the shape of the loops from the rectangular ones. In addition, such films are characterized by significantly lower values of spontaneous magnetization.

In our opinion, to obtain thick epitaxial BaM hexaferrite layers comparable in magnetic characteristics to thin ones, a more complex growth protocol can be used, in which the growth process consists of several stages, each of which includes the growth of a thin (*h*~50 nm) layer and its annealing in air at *T_ann_* = 1000 °C.

## Figures and Tables

**Table 1:** Number of structure, film thickness *h*, growth temperature *T*_gr,_ oxygen pressure *p*, annealing time *t*_ann_, and annealing temperature *T*_ann_.

# Sample Number	Film Thickness *h*, nm	Growth Temperature *T*_gr_, °C	Oxygen Pressure *p*, mBar	Annealing Time *t*_ann_ (min)/Annealing Temperature *T*_ann_, °C
8790	50	850	0.06	no
8939	50	850	0.04	no
8940A	50	850	0.004	no
8940B	50	850	0.004	120/1000
8941A	50	850	0.06	no
8941B	50	850	0.06	30/1000
8944	50	900	0.06	no
8947	50	1000	0.06	no
8948A	50	750	0.06	no
8948B	50	750	0.06	10/1000
8948B^+^	50	750	0.06	20/1000
8948C	50	750	0.06	60/1000
8948C^+^	50	750	0.06	120/1000
8949	50	925	0.06	no
8952 **	50 + 1	800 + 900	0.004	no
8954A	300	750	0.06	no
8954B	300	750	0.06	60/1000
8960A	500	700	0.06	no
8960B	500	700	0.06	180/1000
8960C	500	700	0.06	120/1000
8963A	250	550	0.06	no
8963B	250	550	0.06	60/1000
8963C	250	550	0.06	30/1000
8963D	250	550	0.06	10/1000
9001A	500	700	0.06	60/1000
9001B	500	700	0.06	240/1000
9001C	500	700	0.06	no

** #8952 two-stage sample BFO 50 nm 0.004 mbar O_2_ 800 °C/BFO 0.8 nm 0.004 mbar O2 900 °C/Al_2_O_3_(0001).

**Table 2:** Fe:Ba concentration ratio for films with thickness *h* = 50 nm grown at 750 °C, before (film #8948A) and after annealing at 1000 °C for 10 min (film #8948B).

	As-Grown Sample #8948A		Annealed Sample #8948B	
Point Number	Fe:Ba Mass Ratio	Fe:Ba Atomic Ratio	Fe:Ba Mass Ratio	Fe:Ba Atomic Ratio
1	5.0	12.1	4.1	9.925
2	4. 7	11.2	4.2	10.07
3	5	12	4.0	9.511
4	4.8	11.4	4.8	11.45

**Table 3:** Interplanar spacings *d* and microstructure parameters (average sizes *D*_0_ of crystallites in the model without microstrains (*ε*_s_ = 0), mean sizes *D* of crystallites and absolute mean values of microstrains *ε*_s_ in them) of the observed crystalline phase(s) BaFe_2_O_4_ in unannealed samples #8948A and #9001C. Mean profile type criteria *FWHM*/*B*_int_ averaged over all observed XRD reflection and are shown.

Sample	Interplane Distance *d*, Å	*D*_0_, nm *FWHM*/*B*_int_	WHP *D*, nm *ε_s_*, %	SSP *D*, nm *ε_s_*, %
#8948A ^a^ (without annealing)	0.23813(6) ^b^ 0.11914(1) ^c^	54(28) 0.673(1)	– ^d^ 0.159(8)	– ^d^ 0.159(8)
#9001C ^a^ (without annealing)	0.23736(6) ^b^ 0.11898(1) ^c^	30(11) 0.6721(1)	80(12) 0.22(1)	80(41) 0.22(3)

^a^ Only two reflections are observed, which can be attributed to different orders of one reflection of various modifications of BaFe_2_O_4_ (space group (sp. gr.) *Bb*2_1_*m* (36), *a* = 19.042(4) Å, *b* = 5.3838(7) Å, *c* = 8.4445(7) Å at ambient temperature 298 K, PDF-2 entry 00-046-0113 or sp. gr. *Pmcn* (62), *a* = 17.3469(39) Å, *b* = 9.3358(24) Å, *c* = 10.8818(19) Å at ambient temperature 298 K, PDF-2 entry 01-077-2337); in WHP and SSP graphs, the calculated value of *R*_cod_ = 100% has no physical meaning. ^b^ Interplanar spacing *d* corresponding to the reflection with Miller indices *hkl* = 800 of the BaFe_2_O_4_ modification with sp. gr. *Bb*2_1_*m* (36) or *hkl* = 314 of the BaFe_2_O_4_ phase with sp. gr. *Pmcn* (62). According to the unit cell parameters of the PDF-2 entries 00-046-0113 and 01-077-2337, *d* = 0.23800 Å and 0.23803 Å for phases with space groups *Bb*2_1_*m* (36) and *Pmcn* (62), respectively. ^c^ Interplanar spacing *d* corresponding to the reflection with Miller indices *hkl* = 16 0 0 of the BaFe_2_O_4_ modification with sp. gr. *Bb*2_1_*m* (36) or *hkl* = 628 of the BaFe_2_O_4_ phase with sp. gr. *Pmcn* (62). According to the unit cell parameters of the PDF-2 entries 00-046-0113 and 01-077-2337 *d* = 0.11901 Å and 0.11901 Å for both modifications. ^d^ The graphical line profile analysis technique (WHP or SSP, respectively) evidences the dominant contribution of microstrain to broadening of the observed reflections and no influence of size broadening (“*D* = ∞”).

**Table 4:** Parameters *a* and *c* of the hexagonal unit cell and microstructure parameters (average sizes *D*_0_ of crystallites in the model without microstrains (*ε*_s_ = 0), mean sizes *D* of crystallites and absolute mean values of microstrains *ε*_s_ in them) of the observed crystalline phase BaFe_12_O_19_ ^a^ in annealed samples #8948B, #9001a, and #9001B. Mean profile type criteria *FWHM*/*B*_int_ averaged over all observed XRD reflection and are shown. Coefficients of determination *R*_cod_ of straight WHP (SSP) lines are presented.

Sample	*a*, Å *c*, Å	*D*_0_, nm *FWHM*/*B*_int_	WHP		SSP	
			*R*_cod_, %	*D*, нм *ε_s_*, %	*R*_cod_, %	*D*, нм *ε_s_*, %
#8948B (annealing 10 min)	5.8853(18) 23.120(9)	45(17) 0.69(3)	2.03	45(17) 0	95.22	38(2) 0.03(36)
9001A (annealing 60 min)	5.8618(15) 23.164(6)	53(25) 0.80(10)	4.39	50(8) 0.066(30)	71.95	145(18) 0.173(12)
#9001B (annealing 240 min)	5.8573(45) 23.075(20)	52(23) 0.81(9)	15.06	61(1) 0.117(25)	76.04	100(10) 0.176(17)

^a^ According to PDF-2 00-027-1029 entry of PDF-2, *a* = 5.892 Å, *c* = 23.198 Å at 298 K for BaFe_12_O_19_ (sp. gr. *P*6_3_/*mmc* (194)).

## Data Availability

Data are contained within the article or Supplementary Materials.

## Supplementary Materials

The following supporting information can be downloaded at: https://www.mdpi.com/article/10.3390/ma16124417/s1, Supplementary Materials S1: Powder XRD; Figure S1: (a,c) WHP and (b,d) SSP graphs for the samples (a,b) #8948A and (c,d) #8948B; Figure S2: (a,c,e) WHP and (b,d,f) SSP graphs for the samples (a,b) #9001C, (c,d) #9001A, and (e,f) #9001B. Figure S3: XRD patterns (θ-2θ scans) of the annealed samples #9001A,B and #8948, and α-Al_2_O_3_(0001) substrate. Red lines show positions of hematite α-Fe_2_O_3_ reflections according to PDF-2. References [36,37,38,39,40,41,42,43,44,45] are cited in the Supplementary Materials.
