# Supplementary material for: Structural, Magnetic, and Magneto-Optical Properties of Thin Films of BaM Hexaferrite Grown by Laser Molecular Beam Epitaxy
Source: Materials (Basel). 2023 Jun 15;16(12):4417. doi: 10.3390/ma16124417 (PMC10302779; doi:10.3390/ma16124417)
Supplement: Supplementary file 1 [file materials-16-04417-s001.zip › materials-2398115-supplementary.pdf]

materials

Supplementary Materials

# Structural, Magnetic, and Magneto-Optical Properties of Thin Films of BaM Hexaferrite Grown by Laser Molecular Beam Epitaxy

**Supplementary Materials S1. Powder XRD**

The XRD patterns were analyzed with obtaining the XRD reflection parameters (observed reflection angle positions (Bragg angles $2\theta_B^{obs}$), full width at half maximum (FWHM) *FWHM*, maximum $I_{max}$, and integral $I_{int}$ intensities of the reflections) using the program EVA [1]. Corrections for zero shift $\Delta 2\theta_{zero}$ and displacement $\Delta 2\theta_{displ}$ to obtain corrected Bragg angle values $2\theta_B$ (see [2]) were determined based on additional XRD measurements of samples immersed in NaCl powder calibrated using XRD standard powder Si640f (NIST, Gaithersburg, Maryland, USA), so that the NaCl powder and sample surfaces coincide and are illuminated by the X-ray beam. X-ray-phase analysis of the measured XRD patterns was carried out with the *EVA* program using the Powder Diffraction File-2 database (PDF-2) [3].

Utilizing the corrected Bragg angle values and Miller indices *hkl* of the observed XRD reflections, the unit cell parameters of the crystalline phases of the films were calculated by the least squares method using the program *Celsiz* [4].

The parameters of the microstructure of the films (the mean sizes *D* of the areas of coherent X-ray scattering (crystallites) and the absolute average values $\varepsilon_s$ of the microstrains in them) were determined from FWHM of the XRD reflections using the program *SizeCr* [5] by the Williamson-Hall plot (WHP) [6] and size-strain plot (SSP) [7] methods. The program *SizeCr* uses the procedures, taking into account the type of reflection (Gaussian, Lorentzian, or pseudo-Voigt (pV)), for instrumental correction of the observed *FWHM* values and calculation of *D* and $\varepsilon_s$ from the obtained values of $FWHM_{corr}$.

The type of each XRD reflection with Miller indices *hkl* was determined by the program SizeCr considering the value of the criterion $FWHM/B_{int}$ [8], where $B_{int} = I_{int}/I_{max}$ is the integral width of the reflection. When calculating the WHP and SSP points, the coefficients $K_{strain} = 4$ and $K_{Scherrer} = 0.94$ of the Wilson-Stokes [9] and Scherrer [10] equations were used, respectively, which relate the corresponding contributions to FWHM with the values of $\varepsilon_s$ and *D*.

The mean value $D_0$ of the crystallite size in the model without microstrains ($\varepsilon_s = 0$) and the mean valueD in the case of zero microstrain ($\varepsilon_s = 0$) according to the results of WHP or SSP were calculated by root mean-square (r.m.s.) averaging of the individual values of the sizes $D_{hkl}$ of crystallites obtained from $FWHM_{corr}$ (*FWHM* after correction of instrumental broadening) using the Scherrer equation for each reflection. Similarly, in the absence of a contribution to the FWHM of reflection broadening caused by the size effect (in this case, the size of the crystallite can be considered as "infinite", "$D = \infty$"), the mean value $\varepsilon_s$ of the microstrain was calculated as the r.m.s. average of the individual values of the microstrains $\varepsilon_{s\_hkl}$ obtained for each reflection from $FWHM_{corr}$ value using the Wilson-Stokes equation.

In the WHP and SSP methods, a set of experimental points with coordinates (*X*, *Y*) is plotted on the graphs, where *X* and *Y* are expressions composed of $FWHM_{corr}$, $K_{strain}$, $\cos(\theta_B)$ and/or $\sin(\theta_B)$ in the case of WHP and $FWHM_{corr}$, $K_{Scherrer}$, $\cos(\theta_B)$, interplanar distance *d*, corresponding to the angle $2\theta_B$ according to Bragg's law and the wavelength $\lambda$ of Cu-$K_{\alpha 1}$ radiation (after correcting the contribution of Cu-$K_{\alpha 2}$) in the case of SSP (see [6], [7] or [5], and Figure S1). In both methods, an approximation regression straight line *Y* =

$A + B{\cdot}X$ is drawn on the set of these points, the coefficients of which are calculated by the least squares technique. The mean values of $\varepsilon_s$ and $D$ are calculated, respectively, from the slope $B$ of the approximating straight line and the intersection $A$ of this line with the $Y$ axis in the case of WHP and, on the contrary, from the intersection $A$ of the approximation straight line with the $Y$ axis and the slope $B$ of this line in the case of SSP. Due to experimental inaccuracies in determining the parameters of XRD reflections and/or limitations of the correctness of the model of the WHP and SSP methods, there is a spread of experimental WHP and SSP points around the approximating straight regression lines. This spread results in the standard deviations (e.s.d.s) of the regression coefficients $A$ and $B$ and, respectively, e.s.d.s of the calculated $\varepsilon_s$ and $D$ values. Also, the spread of points around the approximating straight line is characterized by the determination coefficient $R_{cod}$, the expression for which can be found in [5], [7]. The $R_{cod}$ value is greater the smaller the spread of points around the approximating straight line, and $R_{cod}$ = 100% in the ideal case when all experimental points fall on the approximating line.

In the presence of more than two experimental points on the WHP (SSP) graphs, the standard deviations (e.s.d.s) of the values $\varepsilon_s$ and $D$ are calculated analytically using the *SizeCr* program from the r.m.s. e.s.d.s of the regression coefficients $A$ and $B$. If there are only two experimental points, e.s.d.s of the $\varepsilon_s$ and $D$ values are calculated analytically using the *SizeCr* program from the e.s.d.s of the coordinates $X$ and $Y$ of the experimental points of the WHP (SSP) graphs, which, in turn, are calculated analytically from the e.s.d.s of the experimental values of FWHMcorr and 2θB. If there are only two experimental points, the e.s.d.s of the $\varepsilon_s$ and $D$ values are calculated analytically using the *SizeCr* program from the e.s.d.s of the $X$ and $Y$ coordinates of the experimental points of the WHP (SSP) graphs, which, in turn, are calculated analytically from the e.s.d.s of the experimental $FWHM_{corr}$ and $2\theta_B$ values.

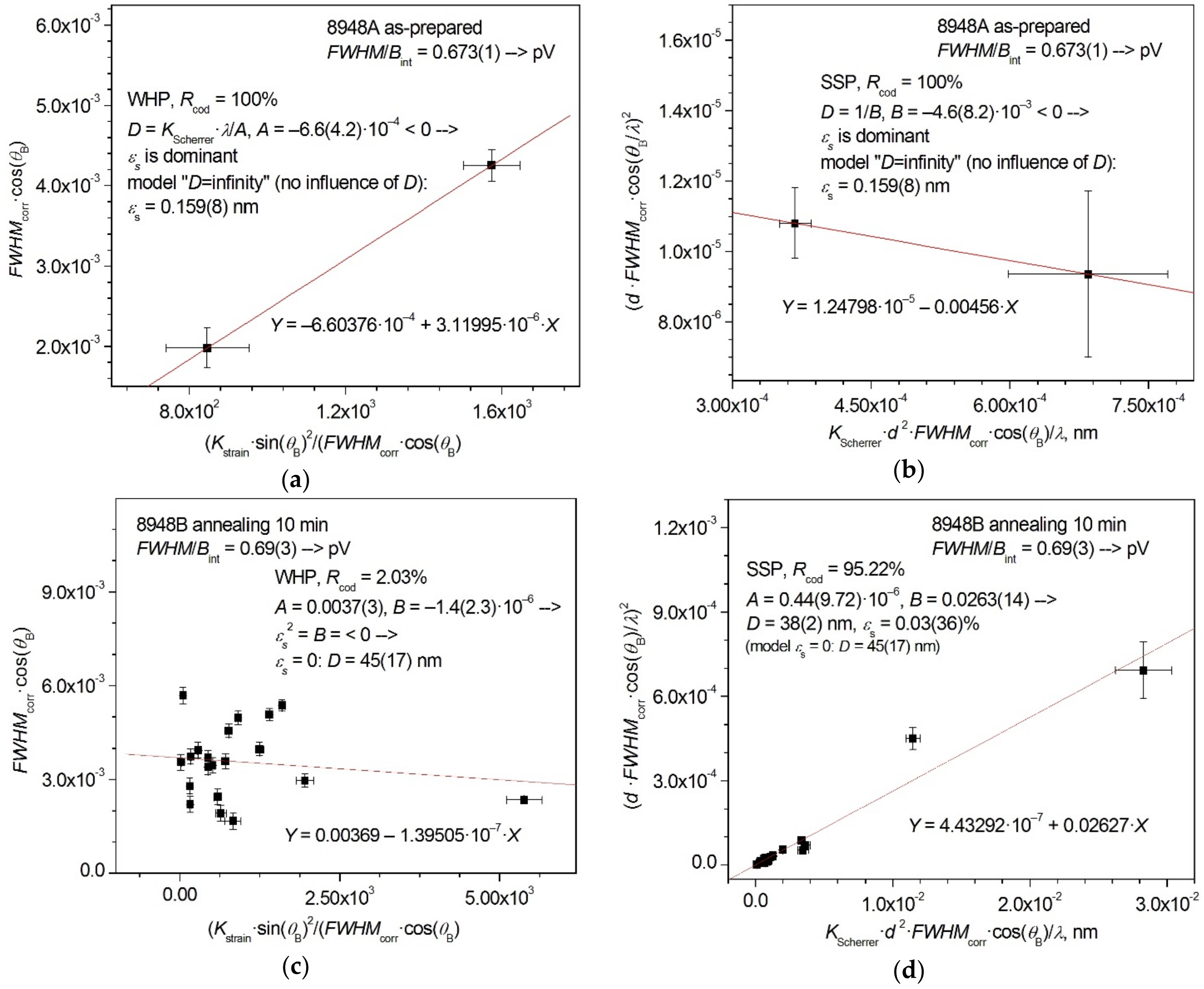


**Figure S1.** (**a**, **c**) WHP and (**b**, **d**) SSP graphs for the samples (**a**, **b**) #8948A as-prepared and (**c**, **d**) #8948D after 10 min annealing.

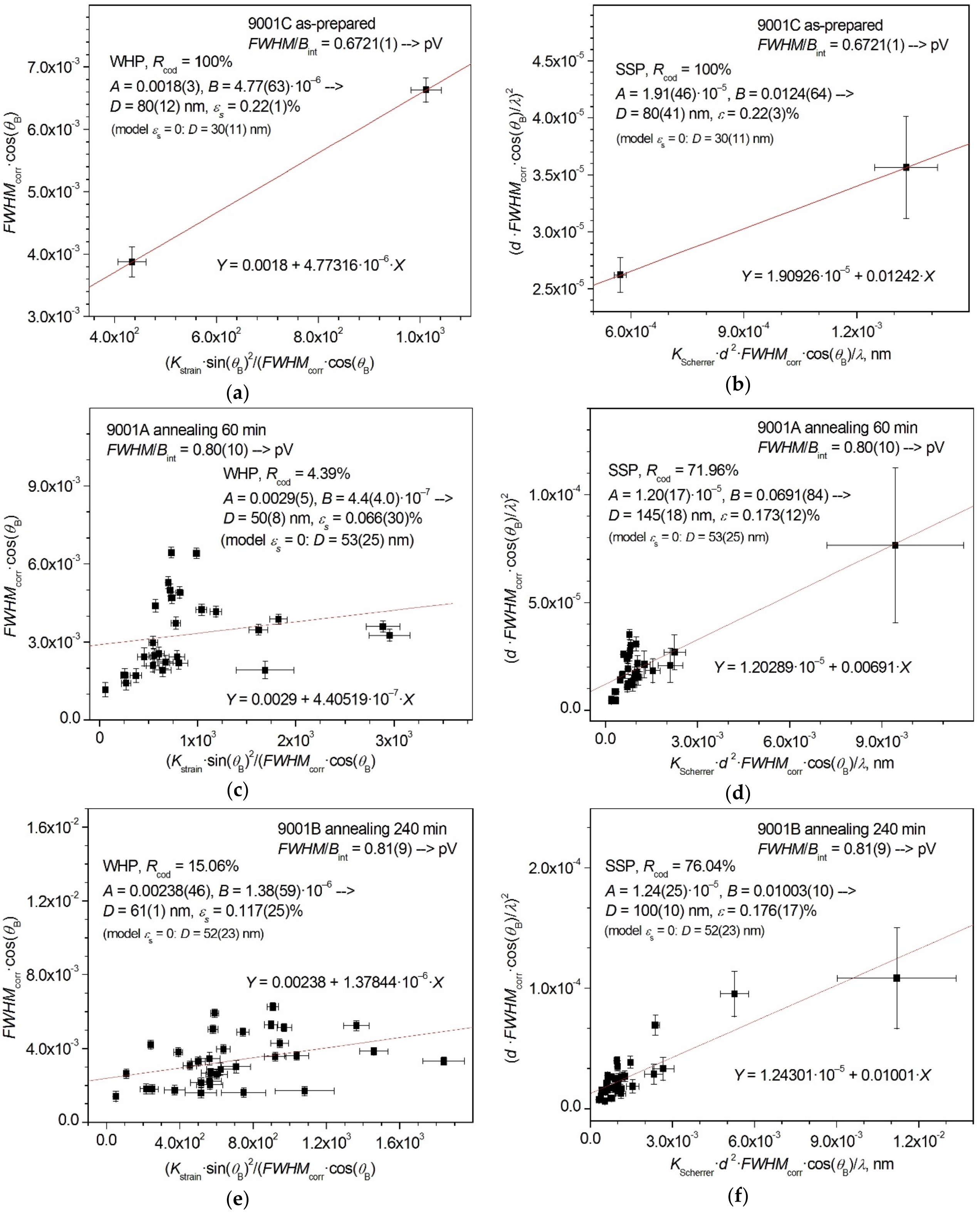


**Figure S2.** (**a**, **c**, **e**) WHP and (**b**, **d**, **f**) SSP graphs for the samples (**a**, **b**) #9001C as-prepared, (**c**, **d**) #9001B after 1 h annealing, and (**e**, **f**) #9001A after 4 h annealing.

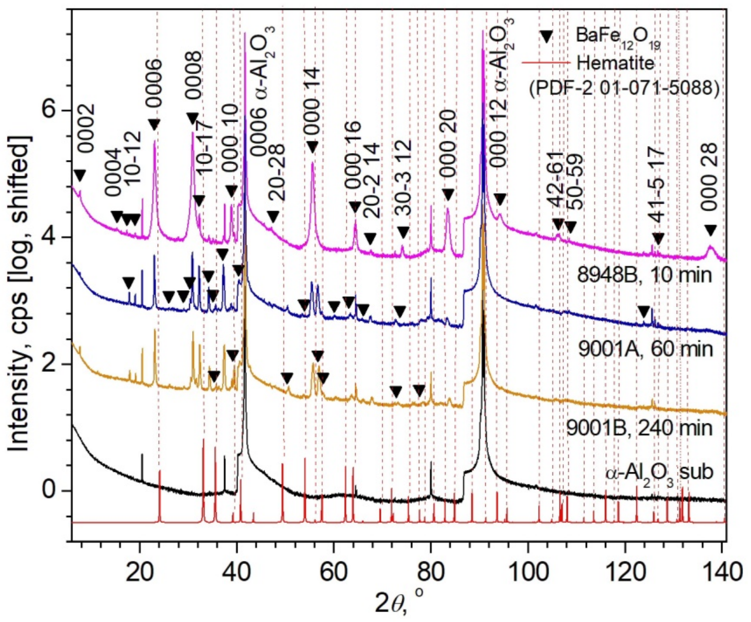


**Figure S3. XRD** patterns ($\theta$-2θ scans) of the annealed samples #9001A,B and #8948, and α-$Al_2O_3$(0001) substrate. Red lines show positions of hematite $\alpha$-$Fe_2O_3$ reflections acoording to PDF-2 card. The Miller-Bravais *hkil* indices of some selected $BaFe_{12}O_{19}$ reflections are indicated. For other information see caption to Figure 6 of the main text of the paper.

Figure S3 shows XRD patterns (θ-2θ scans) of the annealed samples #9001A,B and #8948, and α-$Al_2O_3$(0001) substrate. Red lines shows hematite $\alpha$-$Fe_2O_3$ lines according to PDF-2. All the experimentally observed reflections are well described by the BaM phase. The agreement between the positions of the hematite reflections and the experimental ones is poor. There is no reason to assume the presence of hematite or another phase.

## References


1. *Diffrac. Suite Eva*. Version 5.1.0.5, User Manual, Bruker AXS, Karlsruhe, Germany, 2019.
2. Balashova, E.; Zolotarev, A.; Levin, A. A.; Davydov, V.; Pavlov, S.; Smirnov, A.; Starukhin, A.; Krichevtsov, B.; Zhang, H.; Li, F.; Luo, H.; Ke, H. Crystal Structure, Raman, FTIR, UV-Vis Absorption, Photoluminescence Spectroscopy, TG–DSC and Dielectric Properties of New Semiorganic Crystals of 2-Methylbenzimidazoleium Perchlorate, *Materials* **2023**, *16*, 1994. https://doi.org/10.3390/ma1605199
3. International Centre for Diffraction Data (ICDD). *Powder Diffraction File-2 Release 2014*, ICDD: Newton Square, PA, USA, 2014.
4. Maunders, C.; Etheridge, J.; Wright, N.; Whitfield, H. J. Structure and microstructure of hexagonal $Ba_3Ti_2RuO_9$ by electron diffraction and microscopy. *Acta Crystallogr. B* **2005**, *61*, 154–159. https://doi.org/10.1107/S0108768105001667
5. Levin, A. A. Program SizeCr for calculation of the microstructure parameters from X-ray diffraction data. Preprint, 2022. https://doi.org/10.13140/RG.2.2.15922.89280.
6. Terlan, B.; Levin, A.A.; Börrnert, F.; Simon, F.; Oschatz, M.; Schmidt, M.; Cardoso-Gil, R.; Lorenz, T.; Baburin, I. A.; Joswig, J.-O.; Eychmüller, A. Effect of Surface Properties on the Microstructure, Thermal, and Colloidal Stability of $VB_2$ Nanoparticles. *Chem. Mater.* **2015**, *27*, 5106–5115. https://doi.org/10.1021/acs.chemmater.5b01856
7. Terlan, B.; Levin, A. A.; Börrnert, F.; Zeisner, J.; Kataev, V.; Schmidt, M.; Eychmüller, A. A Size-Dependent Analysis of the Structural, Surface, Colloidal, and Thermal Properties of $Ti_{1-x}B_2$ (x = 0.03–0.08) Nanoparticles, *Eur. J. Inorg. Chem.* **2016**, *6*, 3460–3468. https://doi.org/10.1002/ejic.201600315
8. Langford, J. I.; Cernik, R. J.; Louer, D. The Breadth and Shape of Instrumental Line Profiles in High-Resolution Powder Diffraction. *J. Appl. Phys.* **1991**, 24, 913–919. https://doi.org/10.1107/S0021889891004375
9. Stokes, A. R.; Wilson, A. J. C. The diffraction of X-rays by distorted crystal aggregates, *Proc. Phys. Soc. London* **1944**, *56*, 174−181. https://doi.org/10.1088/0959-5309/56/3/303
10. Scherrer, P. Bestimmung der Grösse und der inneren Struktur von Kolloidteilchen mittels Röntgenstrahlen, *Nachr. Königl. Ges. Wiss. Göttingen*. **1918**, *26*, 98–100 (in German)
